# The complete plastid genome and phylogenetic analysis of *Gracilaria textorii*

**DOI:** 10.1080/23802359.2019.1642163

**Published:** 2019-07-17

**Authors:** Weizhou Chen, Tao Liu, Xianming Tang, Xuli Jia, Xiangyu Wu

**Affiliations:** a Marine Biology Institute, Shantou University, Shantou, People’s Republic of China;; b Hainan Academy of Ocean and Fisheries Sciences, Haikou, People’s Republic of China;; c Hainan Provincial Key Laboratory of technology for tropical seawater aquaculture, Haikou, People’s Republic of China;; d Laboratory of Genetics and Breeding of Marine Organism, College of Marine Life Sciences, Ocean University of China, Qingdao, People’s Republic of China

**Keywords:** *Gracilaria textorii*, Gracilariaceae, plastid genome, phylogenetic analysis

## Abstract

The complete plastid genome of *Gracilaria textorii*, a marine red macroalga, was determined and analyzed. The plastid genome sequence of *G. textorii* is 179,609 bp. It contains 237 genes, including 203 protein-encoding genes, 30 tRNA genes, 3 rRNA genes, 1 ribonuclease gene, and 1 intron inserted into the *trnM* gene. Phylogenetic analysis showed that *G. textorii* clustered together with *Gracilaria salicornia*, which helps the better understanding of *Gracilaria* evolution process.

*Gracilaria textorii* (Suringar) De Toni is a marine red alga belonging to the family Gracilariaceae (http://www.algaebase.org/). *Sphaerococcus textorii* Suringar is the basionym of *G. textorii*. It is a taurine-rich red edible alga which may be used to create functional foods that are rich in naturally occurring taurine. (Kawasaki et al. 2017). The seaweed extract has the anti-inflammatory effect (Kim et al. 2018). It also was found effective on the germination, growth, and yield of some vegetable crops which can be used as fertilizer. In addition, a quantitative differential gene expression analyses of some *G. textorii* genes indicated this species could be applicable to marine environment monitoring (Woo et al. 2008). However, until recently, no genomic studies on *G. textorii* have been reported.

The determination of the complete *G. textorii* plastid genome sequence by next-generation sequencing methods was conducted. The specimen was collected from north China (Taigong Island, Rizhao, Shandong Province, 35°27′54′′ N, 119°35′58′′ E) and stored at the Culture Collection of Seaweed at the Ocean University of China (accession number: 2017050065). Total DNA was extracted using the modified CTAB method (Doyle and Doyle 1990). Paired-end reads (150 bp) were sequenced by using Illumina HiSeq system (Illumina, San Diego, CA, USA). tRNAscan-SE Search Server (Schattner et al. 2005) was used to identify the tRNA genes. The other plastid genomic regions were annotated with Geneious R10 (Biomatters Ltd, Auckland, New Zealand), using the *Gracilaria chilensis* (NC_029860) plastid genome as a reference.

The complete *G. textorii* plastid genome is a circular DNA molecule measuring 179,609 bp in length with the overall G + C content of 28.78% (GenBank accession number MN053320). The plastid genome contained 237 genes, including 203 protein-coding genes, 1 ribonuclease gene (*rnpB*), 3 rRNA genes, 30 tRNA genes, and 1 intron interrupting the *trnM* gene. The nucleotide composition was 35.73% A, 14.44% C, 14.34% G, and 35.49% T. The length of the coding region was 145,209 bp, corresponding to 80.85% of the total length. The plastid genome of *G. textorii* was compact, with 11 pairs of overlapping genes found with overlap lengths of 3–95 bp (*trnT*–*ilvB*, *rnpB*–*syfB*, *ycf40*–*rps1*, *ycf29–trnH*, *psbD*–*psbC*, *carA*–*ycf53*, *trnR*–*chlI*, *atpF*–*atpD*, *rps18*–*rpl33*, *rpl23*–*rpl4*, and *rpl14*–*rps17*).

82 shared plastid protein sequences from 17 red algae including *G. textorii* were used to conduct phylogenetic analysis by using MrBayes 3.1.2 software (Ronquist and Huelsenbeck 2003). *Cyanidioschyzon merolae* (NC_004799) was served as the outgroup. Poorly aligned regions were removed by using the Gblocks server. Florideophyceae, Bangiophyceae and Cyanidiophyceae species formed separate branches (Figure 1). *G. textorii* showed a closer relationship with *Gracilaria salicornia* in *Gracilaria*. This complete plastid genome analysis of *G. textorii* helps us better understand the evolutionary process of *Gracilaria*.

**Figure 1.**
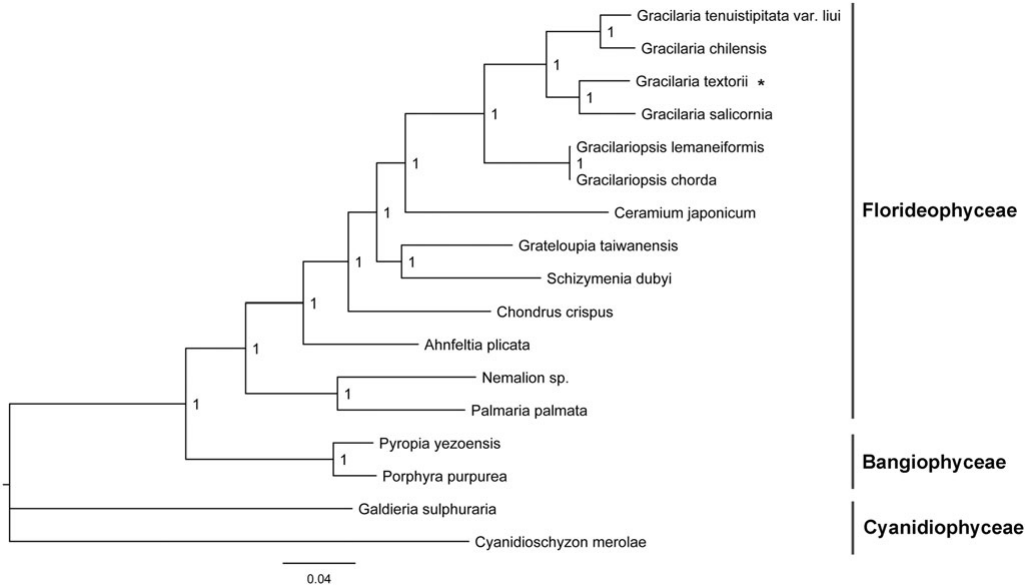
Phylogenetic tree (Bayesian method) based on the complete plastid genome sequence of red algae as shown below: *Gracilaria textorii* (MN053320), *Gracilaria salicornia* (NC_023785), *Gracilaria tenuistipitata* var. *liui* (AY673996), *Gracilaria chilensis* (NC_029860), *Gracilariopsis chorda* (NC_031149), *Gracilariopsis lemaneiformis* (KP330491), *Grateloupia taiwanensis* (KC894740), *Schizymenia dubyi* (NC_031169), *Chondrus crispus* (NC_020795), *Ceramium japonicum* (NC_031174), *Nemalion* sp. (LT622871), *Ahnfeltia plicata* (NC_031145), *Palmaria palmata* (NC_031147), *Pyropia yezoensis* (KC517072), *Porphyra purpurea* (U38804), *Galdieria sulphuraria* (KJ700459), and *Cyanidioschyzon merolae* (NC_004799). The asterisks after species names indicate newly determined plastid genomes.
